# A Novel Model of Meibomian Gland Dysfunction Induced with Complete Freund’s Adjuvant in Rabbits

**DOI:** 10.3390/vision1010010

**Published:** 2017-02-09

**Authors:** Hideki Miyake, Tomoko Oda, Osamu Katsuta, Masaharu Seno, Masatsugu Nakamura

**Affiliations:** 1 Research and Development Division, Santen Pharmaceutical Co., Ltd., Osaka 5308552, Japan; 2 Department of Medical Bioengineering, Division of Medical Bioengineering, Graduate School of Natural Science and Technology, Okayama University, Okayama 7008530, Japan

**Keywords:** plugging, inflammation, steroid, antibiotics

## Abstract

A novel meibomian gland dysfunction (MGD) model induced by the injection of complete Freund’s adjuvant (CFA) in rabbits was developed to facilitate the understanding of the pathophysiology of MGD with meibomitis. In addition, we sought to evaluate treatment with steroid eye drops in this model. Male Japanese white rabbits were subcutaneously injected with CFA into the upper eyelid margin. The eyelid margins of the rabbits were chronologically observed through slit lamp examination. The development of meibomitis was assessed through histopathology. We evaluated the effects of topically applied tobramycin/dexamethasone (Tob/Dex) eye drops on the plugged orifices and telangiectasia. After the injection of CFA, slit lamp examination revealed markedly plugged orifices, telangiectasia around the orifices and a toothpaste-like meibum, as compared with the normal eyelids. Histopathology revealed granulation tissue with infiltration of inflammatory cells, hyperkeratinization of the ductal epithelium, and cystic dilatation of ducts in the meibomian gland. The orifices were plugged with a proteinaceous substance. Tob/Dex eye drops significantly suppressed the plugging and telangiectasia around the orifices. Through the injection of CFA, we successfully established a novel rabbit MGD that mimics the symptoms observed in humans meibomitis. This model should be useful in the evaluation of the efficacy of drug candidates.

## 1. Introduction

The meibomian glands are a type of sebaceous gland that is vertically arranged within the upper and lower tarsal plates [1]. These glands secrete lipids that form a superficial oily layer on the tear film. Meibomian gland dysfunction (MGD) is defined as a chronic and diffuse abnormality of the meibomian glands and is commonly observed with terminal duct obstruction and/or qualitative or quantitative changes in glandular secretion [2]. MGD results in alteration of the tear film, eye irritation, clinically apparent inflammation, and ocular surface disease [3]. The pathogenesis of both MGD and dry eye disease (DED) has recently been described in terms of a “vicious cycle”, wherein the underlying pathophysiological mechanisms of DED and MGD interact, thus resulting in pronounced clinical effects [4].

In patients with characteristic signs of obstructive MGD, slit lamp microscopy has revealed that the meibomian gland orifices are closed by plugs of thickened and opaque secretions containing keratinized material with telangiectasia around the orifice and eyelid margin rounding [1,5]. Furthermore, MGD patients experience impaired quality of both life and vision because the oily layer plays an important role in maintaining the stability of the tear film by preventing tear evaporation and reducing tear surface tension and the friction of blinking [5]. However it is controversial whether the oily layer reduces tear evaporation [6]. Given the increasing recognition of the importance of MGD, considerable attention has been paid to therapies targeting this condition. Traditional MGD treatments consist of warm compresses and eyelid hygiene to remove the obstructed meibum [7,8,9]. Some pharmacological therapies such as azithromycin, doxycycline and others show the efficacy through the improvement of meibum quality in MGD patients [7,10,11]. However, no pharmacological therapy for MGD has been approved to date. Thus, the causes and progression of MGD require further investigation to develop better pharmacologic treatments.

Both systemic and local factors may contribute to the pathogenesis of MGD. We have previously developed a novel MGD model induced by feeding a special diet containing limited lipid content to HR-1 hairless mice to demonstrate the characteristic clinical signs and atrophy of acinar cells in meibomian glands. Moreover, the switch to a normal diet ameliorated the plugged orifices and restored the tissue to normal [12]. Our data revealed that inflammation was not critical for the development of MGD in this model. 

However, the relevance of inflammation in the pathophysiology of MGD remains controversial [13], Suzuki et al. [14] have proposed that MGD is divided into two main types: (1) inflamed/obvious and (2) non-inflamed/non-obvious, and that meibomitis is inflamed obstructive MGD. Topical loteprednol etabonate and eyelid scrubs with warm compresses have been found to improve clinical outcomes, tear film break-up time (TBUT), corneal and conjunctival fluorescein staining, and meibum quality [15], thus indicating that inflammation plays a considerable role in the onset of MGD.

Appropriate animal models mimicking the pathogenesis of MGD with inflammation observed in humans are strongly desirable to understand the pathophysiology of the disease and to aid in development of potential pharmacologic interventions. There are several spontaneous genetic animal models [16,17,18,19,20,21,22] and chemically induced models of MGD [23,24,25]. Although these models suggest potential underlying causes and key molecular events of the disease, the roles of bacterial components and inflammation in the development of MGD remain unknown. Indeed, the lack of an appropriate model is a major reason for the lack of pharmacologic treatments for MGD.

In this paper, we demonstrate the development of novel MGD with meibomitis model by injecting complete Freund’s adjuvant (CFA) containing heat-killed *Mycobacterium tuberculosis* and paraffin oil in rabbits. Simultaneously, we describe the pathology of MGD together with the temporal morphological changes in inflamed meibomian gland and the efficacy of Tobramycin/dexamethasone ophthalmic suspension (Tob/Dex) in the treatment using this model.

## 2. Results

### 2.1. MGD with Meibomitis Assessed through Slit Lamp Examination

**Figure 1:**
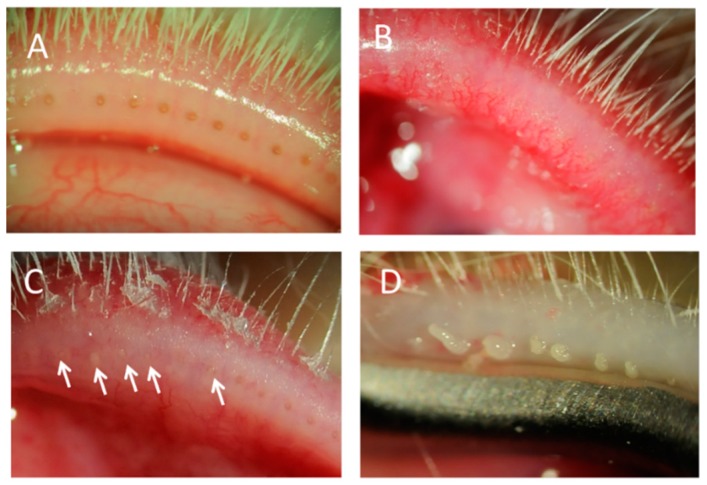
Eyelids of rabbit meibomian gland dysfunction (MGD) with meibomitis model. Slit lamp photographs of the eyelid margins injected with saline (**A**) or complete Freund’s adjuvant (CFA) (**B**–**D**). Rounding, plugging (arrows) and telangiectasia at day 15 (**B**,**C**) and a toothpaste-like meibum at day 21 (**D**) in CFA-injected rabbits.

Figure 1 shows photographs of the eyelid margin under a slit lamp. A marked difference was observed at the eyelid margins between normal control (Figure 1A) and CFA-injected eyes (Figure 1B–D). Telangiectasia around the orifices, palpebral conjunctiva hyperemia (Figure 1B), and many plugged orifices (Figure 1C, arrowheads) were observed in the eyelid margins. Slit lamp examination 4 days after the injection indicated that these changes surrounding the orifices progressed as the period after injection was extended. Thickened secretions were discharged, and a toothpaste-like meibum appeared at day 21 (Figure 1D) while no changes in the eyelid or eyelid margin were observed in rabbits injected with saline (Figure 1A). The central and temporal sites showed more severe inflammation and more plugged orifices than the nasal site (Table 1).

### 2.2. Histopathologic Analysis of MGD with Meibomitis

**Figure 2:**
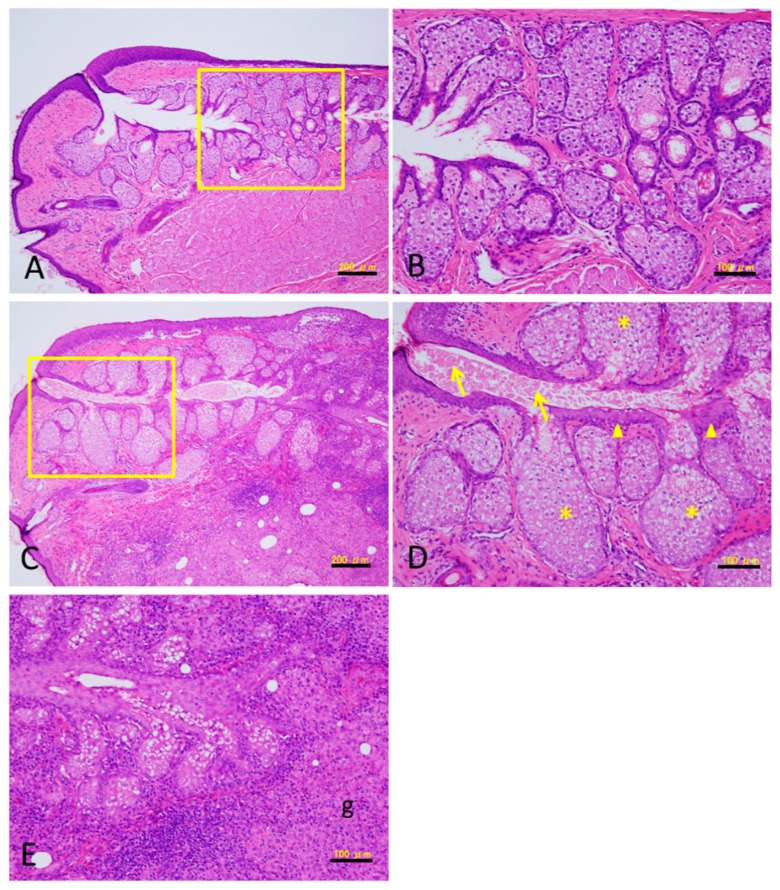
Hematoxylin-enosine (HE) staining of the sections of meibomian gland. Twenty-nine days after saline injection (**A**,**B**) and CFA injection (**C**–**E**). (**B**,**D**) show higher-power views within the rectangular enclosure in (**A**,**C**), respectively. (**E**) shows damaged glands by granulomatous inflammation (g). Thickening and hyperkeratinization of the ductal epithelium (arrowheads in **D**). Plugging with a proteinaceous substance (arrows in **D**). Hypertrophy and/or hyperplasia in the acinar cells (asterisks in **D**). Bar = 100 µm (**B**,**D**,**E**), 200 µm (**A**,**C**).

We evaluated histologic changes in the nasal, central and temporal portions of the eyelid after CFA injection. Figure 2 presents the Hematoxylin-enosine (HE) staining results for the central portion in the eyelid. CFA injection induced granulomatous inflammation with destruction of the meibomian glands (Figure 2C,E). The eyelids also exhibited thickening and hyperkeratinization of the ductal epithelium and dilation of the duct in the meibomian glands (Figure 2C,D arrowheads). Proteinaceous substances accumulated in the ducts and plugged the orifices (Figure 2D arrows). In addition, acinar cells became hypertrophic and/or hyperplastic (Figure 2D asterisks). These changes were identical for each portion of the eyelid. However, the meibomian glands in the rabbits injected with saline did not exhibit any changes throughout the experimental period (Figure 2A,B).

### 2.3. Treatment with Tob/Dex

At each site with CFA injection, the telangiectasia score and plugged orifice score increased up to 11 days after randomization (Table 1).

**Figure 3:**
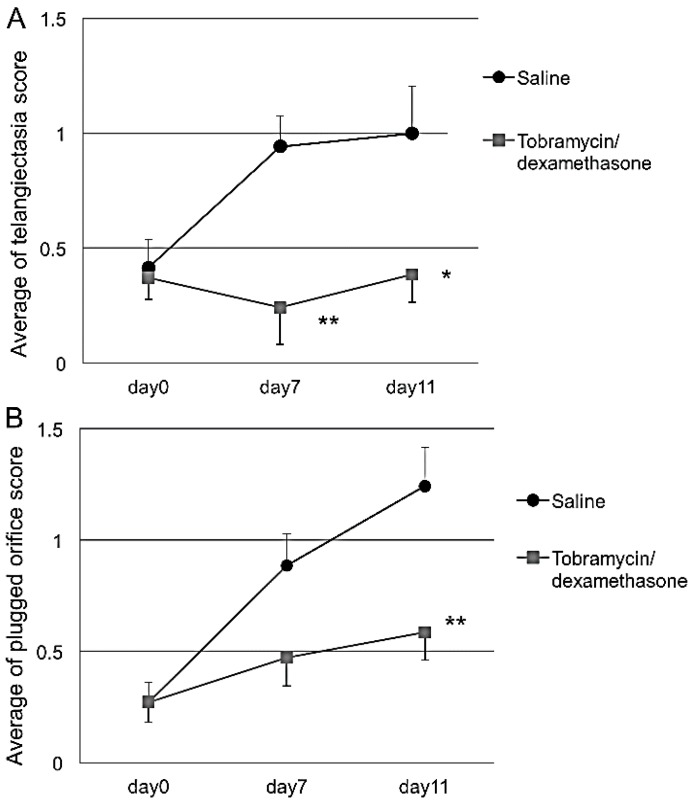
Effect of Tob/Dex on the telangiectasia (**A**) and plugged orifice (**B**). CFA-induced MGD with meibomitis in rabbits was treated with the drug for 11 days. The scores were monitored in the presence or absence of the drug. The data express the average score of three regions of the upper eyelid. Tob/Dex eye drops significantly suppressed the increase in both scores in the telangiectasia and plugged orifice. Each bar represents the mean ± S.E. of 7 eyes. *: *p* < 0.05, **: *p* < 0.01 vs. saline (Student’s *t*-test).

**Figure 4:**
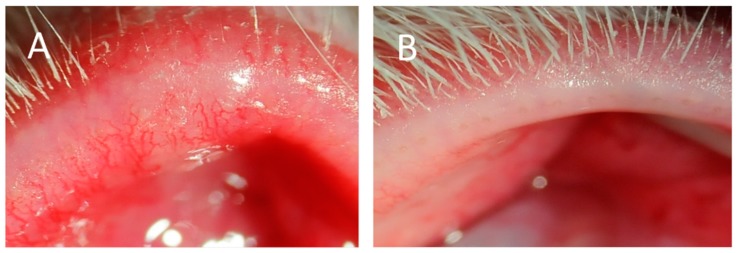
Slit lamp photographs of the eyelid margins injected with CFA. CFA-induced MGD with meibomitis in rabbits was treated with saline (**A**) and Tob/Dex (**B**) for 11 days.

Topically applied Tob/Dex significantly suppressed the increase in the telangiectasia score for the temporal, central and whole eyelid sections on days 7 and 11. The plugged orifice score was also significantly low for the temporal, central and whole eyelid sections on day 11 (Table 1; Figure 3A,B). Typical clinical photograph provided evidence that plugging and telangiectasia was almost cured by the Tob/Dex (Figure 4B) when compared with that treated with saline (Figure 4A) for 11 days.

## 3. Discussion

MGD is a chronic and diffuse abnormality of the meibomian glands. Gram-positive bacteria, such as Coagulase-negative Staphylococcus, Propionibacterium acnes and Cornyneform bacteria, are the most common bacteria isolated from the eyelids of MGD patients as well as of healthy humans [26,27,28,29]. However, the role of commensal bacteria in the development of MGD remains unclear. It has been reported that some species of gram-positive bacteria in eyelids of MGD are significantly present more often than those in healthy eyelids [30]. It is possible that innate immunity without tolerance is essential for the pathogenesis of MGD. In addition, Suzuki et al. have proposed a new disease subset termed meibomitis-related keratoconjunctivitis (MRKC) [14]. Meibomitis is thought to be an inflammatory form of MGD, and the primary clinical feature of MRKC is the occurrence of meibomitis, which is defined as stagnation of meibum and swelling of the eyelid margin and palpebral conjunctiva hyperemia, and particularly telangiectasia around the meibomian gland orifices.

We successfully developed a novel MGD model with meibomitis induced by CFA injection in rabbits. CFA containing heat-killed Mycobacterium tuberculosis and paraffin oil will form a viscous water-in-oil emulsion when mixed with aqueous solutions. Injection of the emulsion induces chronic inflammation, such as the infiltration of inflammatory cells and cell-mediated immunity. However, macroscopic and histopathologic observations of meibomian glands in CFA-injected rabbits have not been reported to date. In this study, CFA injection exhibited the early onset of characteristic clinical signs of MGD with meibomitis and cystic dilation of ducts and granulation tissue in the meibomian glands. This study also revealed that plugged orifices, palpebral conjunctiva hyperemia, telangiectasia surrounding the orifices, and a toothpaste-like meibum were observed under slit lamp examination in rabbits. Because the vast majority of MGD associated with dry eye is rather characterized by MG dropout due to plugging and acinar atrophy without severe inflammation, our novel model may mirror the pathophysiology of a meibomitis as a subtype of human MGD [14]. 

Histological examination of the eyelids revealed that CFA induced inflammatory cell infiltration, ductal epithelial hyperkeratinization, granulation, meibomian hypertrophy, hyperplasia and the plugged orifices with proteinaceous substance. Granulation tissue is an indicator of a chronic inflammatory response. Obata has reported the histopathology of cystic dilatation of acini and/or ducts and granulation tissue in the meibomian gland in aging subjects [31]. Samples of abnormal human meibum collected from MGD patients exhibited elevated level of total protein [32], and increase of keratins/cytokeratins [33,34]. Viscous ordered lipids blocked the ducts sometimes followed by the proteinaceous conjunction [35]. Our present model resembles the observations reported in human subjects.

However, histological data in our current model did not reveal atrophy of acinar cells, whereas acinar atrophy was observed in the specimens of human meibomian gland. The meibum component, blink rate and TBUT in rabbits are so different from those of humans that these facts should be noted when biochemistry and biophysics of the meibomian lipid are studied to clarify the relationship between MGD with meibomitis and evaporative-type dry eye in humans [36,37,38].

In the model of experimental systemic lupus erythematosus (SLE) reported by Chan et al., the histology of the eyelids shows marked infiltration of polymorphonuclear neutrophils, macrophages, and lymphocytes [39]. In addition, the meibomian glands in this model exhibit varying stages of hypertrophy and mild hyperplasia with inflammation. Our findings also suggest the possibility of the development of hypertrophy and hyperplasia as a compensatory response to the loss of acinar cells due to granulomatous inflammation or as a regenerative process for tissues injured by chronic inflammation. In future studies, we will investigate the mechanism of hypertrophy and hyperplasia in detail and the subtype of lymphocytes involved.

Tob/Dex is used as a combination steroid and antibiotic eye drop indicated for acute anterior blepharitis. This formulation is often used for posterior blepharitis [13]. However, the functional role of topical corticosteroids in the effect on MGD is controversial because inflammation is not clearly identified in MGD [13]. In this study, Tob/Dex suppressed not only telangiectasia but also plugging of the orifice. Steroid eye drops could be one of the options to treat MGD and meibomitis, but the chronic use should be refrained to avoid the side effects. 

## 4. Materials and Methods

### 4.1. Animals

Japanese white rabbits weighing 1.50 to 1.99 kg were obtained from Kitayama Labes Co., Ltd. (Ina, Nagano, Japan). The rabbits were housed under a 12-h light-dark cycle (light on at 7:00 a.m.) at room temperature (23 ± 1 °C) and a humidity of 55% ± 10% and were given ad libitum access to tap water and a gamma-ray sterilized pellet diet (LRC4; Oriental Yeast Co., Ltd., Tokyo, Japan) of approximately 130 g/day. 

### 4.2. Induction of the MGD with Meibomitis Model

The MGD with meibomitis model was induced with CFA containing killed Mycobacterium tuberculosis, strain H37Ra (Difco Lab, Detroit, MI, USA). Under topical anesthesia (Benoxil ophthalmic solution 0.4%, Santen, Osaka, Japan), CFA (each 10 µL) was injected into the nasal, central and temporal upper eyelid margin of the right eye. Saline was injected in the left eye as a control. The experimental procedure was performed in accordance with the guidelines of the Association for Research in Vision and Ophthalmology (ARVO) concerning the use of animals in ophthalmic and vision research. All of the experimental procedures were approved by the Committee on Animal Research at Santen Pharmaceutical Co., Ltd (Osaka, Japan).

### 4.3. Slit Lamp Examination

The development of MGD with meibomitis was evaluated on the basis of the presence of plugged orifices and telangiectasia. The meibomian gland orifices of rabbits were assessed under a slit lamp (SL-D7; Topcon, Tokyo, Japan) and were imaged using a digital camera (D300s; Nikon, Tokyo, Japan). All images were obtained using the same camera with the same settings. Since the conditions of inflammation and plugged orifices appeared variable depending on each region, the numbers of plugged orifices were separately scored in the nasal, central and temporal regions of the upper eyelid as follows: 0, none; 1, less than 4 plugged orifices; 2, greater than 4 and less than 7 plugged orifices; and 3, greater than 7 plugged orifices. Nine was the maximal total score for each region. In this study, plugged orifices were defined as opaque and swollen meibomian gland orifices.

The telangiectasia intensity around the orifice was separately scored in the nasal, central and temporal regions of the upper eyelid as follows: 0, none; 1, mild; 2, moderate; and 3, severe. Nine was the maximal total score for each region. 

### 4.4. Histologic Analysis

Eyelid tissue from the nasal, central and temporal portions of the eye, which included the meibomian gland orifices, was dissected at day 29. The tissue was fixed with 10% neutral buffered formalin solution, embedded in paraffin, and vertically cut into 2-μm-thick sections. The sections were stained with HE for light microscopic examination. 

### 4.5. Treatment of MGD with Meibomitis

Rabbits were randomized into two groups 4 days after the CFA injection. Saline or tobramycin (0.3%) and dexamethasone (0.05%) ophthalmic suspension (TobraDex^®^ST, Alcon Laboratories, Inc., Fort Worth, TX, USA) was instilled by 50 µL/eye four times daily into the right eye from day 5 to day 15. The plugged orifices and telangiectasia were scored in the same manner as described above on days 7 and 11 after randomization. 

### 4.6. Statistical Analysis

Data are expressed as the means ± S.E. The statistical significance of the differences was assessed using Aspin-Welch tests or Student’s *t*-tests. *p*-values less than 0.05 were considered to be statistically significant.

## 5. Conclusions

To our knowledge, this is the first report to demonstrate the peripheral inflammation induced in the eyelid by CFA containing bacterial components leads to meibomitis as a subtype of obstructive MGD. In addition, steroid/antibiotics eye drops exhibit significant efficacy in alleviating the symptoms. This model should help the development of new treatments elucidating the pathogenesis of MGD with meibomitis on a molecular basis.

## Figures and Tables

**Table 1:** Change of telangiectasia score and plugged orifice score with treatment of tobramycin/dexamethasone.

Treatment	Days after randomization	Telangiectasia Score (Mean ± SE)				Plugged Orifice Score (Mean ± SE)			
		Temporal	Central	Nasal	Total	Temporal	Central	Nasal	Total
Saline	0	0.3 ± 0.2	0.7 ± 0.2	0.3 ± 0.2	1.3 ± 0.4	0.1 ± 0.1	0.6 ± 0.2	0.1 ± 0.1	0.9 ± 0.3
	7	1.3 ± 0.3	1.3 ±0.2	0.3 ± 0.2	2.9 ± 0.4	0.9 ± 0.1	1.3 ± 0.3	0.4 ± 0.3	2.6 ± 0.4
	11	1.1 ± 0.3	1.4 ±0.3	0.4 ± 0.2	3.0 ± 0.6	1.1 ± 0.1	1.9 ± 0.1	0.7 ± 0.4	3.7 ± 0.5
Tobramycin/Dexamethasone	0	0.0 ± 0.0	1.0 ±0.2	0.1 ± 0.1	1.1 ± 0.3	0.1 ± 0.1	0.7 ± 0.2	0.0 ± 0.0	0.9 ± 0.3
	7	0.3 ± 0.2 *	0.3 ±0.2 **	0.1 ± 0.1	0.7 ± 0.5 **	0.6 ± 0.3	0.7 ± 0.2	0.1 ± 0.1	1.4 ± 0.4
	11	0.4 ± 0.2	0.6 ± 0.2 *	0.1 ± 0.1	1.1 ± 0.3 *	0.6 ± 0.2 *	0.9 ± 0.3 **	0.3 ± 0.2	1.7 ± 0.4 **

Note: *n* = 7, *: *p* < 0.05, **: *p* < 0.01 compared with Saline groups at each time point (Student’s *t*-test).
